# An Artificial Piece for an Ear

**Published:** 1898-05

**Authors:** William Dunn

**Affiliations:** Florence, Italy


### AN ARTIFICIAL PIECE FOR AN EAR.

BY WILLIAM DUNN, D.D.S., FLORENCE, ITALY.

AMONG the curious and unusual cases which the dental practitioner is sometimes called upon to undertake the following may present some interesting features. The illustrations from photos speak for themselves. The patient, a young man of twenty-four years, while out shooting, had the misfortune to discharge his gun a little too close to his head while jumping a fence, and blew off a piece of his left ear. The wound healed slowly and left a permanent deformity which was very noticeable when standing full face, and much distressed the patient, who saw his chances as a suitor decidedly on the wane. He was sent to me by an orthopædic doctor, after vain attempts at patching him up.

The course I followed was the simple one of building up the missing part in modelling compound as much as was possible, making a vulcanite piece in the usual way, then building up other parts on it again with modelling compound and revulcanizing. It was a work of patience, for there were many inequalities on the piece, and great difficulty was experienced in getting close adaptation at the edges, the soft tissue of the ear giving way under the slightest pressure. Springs and clips were tried and abandoned, not being tolerated by the very delicate skin back of the ear. The solid and somewhat extensive piece, as shown in the illustration, was found to be the only practicable one. When complete, the vulcanite was painted with oil color in close imitation of the skin. Powdered talc and very fine pumice removed the gloss of the dried paint; and the edges were lined with cotton-wool attached with collodion on the under surface, to give the appearance of soft down and make the edges less visible. The completed work presented a very life-like appearance, has not been at all noticeable, and has given satisfaction for over a year. Photos were taken with apparatus kindly lent by Mr. Longworth Powers, son of the great Hiram Powers.

